# The action of Con-ikot-ikot toxin on single AMPA-type glutamate receptors

**DOI:** 10.1085/jgp.202112912

**Published:** 2022-04-04

**Authors:** Jelena Baranovic, Sebastian Braunbeck, Nikolai Zaki, Sonja Minniberger, Miriam Chebli, Andrew J.R. Plested

**Affiliations:** 1 Leibniz Forschungsinstitut für Molekulare Pharmakologie, Berlin, Germany; 2 NeuroCure, Charité Universitätsmedizin, Berlin, Germany; 3 Institute of Biology, Cellular Biophysics, Humboldt Universität zu Berlin, Berlin, Germany; 4 University of Edinburgh, School of Biological Sciences, Edinburgh, UK

## Abstract

Conotoxins are a large group of naturally occurring toxic peptides produced by the predatory sea snails of the genus *Conus*. Many of these toxins target ion channels, often with high specificity and affinity. As such, they have proven to be invaluable for basic research, as well as acting as leads for therapeutic strategies. Con-ikot-ikot is the only conotoxin so far identified that targets AMPA-type glutamate receptors, the main mediators of excitatory neurotransmission in the vertebrate brain. Here, we describe how the toxin modifies the activity of AMPA receptors at the single-channel level. The toxin binds to the AMPA receptor with *EC*_50_ of 5 nM, and once bound takes minutes to wash out. As shown previously, it effectively blocks desensitization of AMPA receptors; however, compared to other desensitization blockers, it is a poor stabilizer of the open channel because toxin-bound AMPA receptors undergo frequent brief closures. We propose that this is a direct consequence of the toxin’s unique binding mode to the ligand-binding domains (LBDs). Unlike other blockers of desensitization, which stabilize individual dimers within an AMPA receptor tetramer, the toxin immobilizes all four LBDs of the tetramer. This result further emphasizes that quaternary reorganization of independent LBD dimers is essential for the full activity of AMPA receptors.

## Teaser

Baranovic et al. describe how Con-ikot-ikot conotoxin modulates the activity of AMPA receptors, major mediators of excitatory neurotransmission. Con-ikot-ikot blocks AMPA receptor desensitization, like cyclothiazide and (R, R)-2b, but unlike them it is a poor stabilizer of the fully open channel.

## Introduction

Predatory sea snails of the genus *Conus* are a rich source of toxic peptides known as conotoxins. Conotoxins are mostly disulfide-rich peptides that target invertebrate and vertebrate ion channels with high specificity. With >500 species of *Conus* snails and each snail producing a venom composed of >100 different peptides, there are more than 50,000 different, pharmacologically active conotoxins (Terlau and Olivera, 2004). This vast natural pharmacy has proven vital for basic research on ion channels (Safo et al., 2000; Xiong et al., 2020), as well as for the development of various therapeutics (Essack et al., 2012). Ziconotide is the first FDA-approved drug based on a conotoxin and is used in the treatment of severe chronic pain. A number of conotoxin-based drugs are currently in clinical trials for various pathologies, such as a peptide based on conantokin G, a conotoxin that targets NMDA receptors, for the treatment of epileptic seizures (Essack et al., 2012). The majority of conotoxins target voltage and ligand-gated ion channels, with acetylcholine receptors the most common target among the latter.

**Figure 1.**
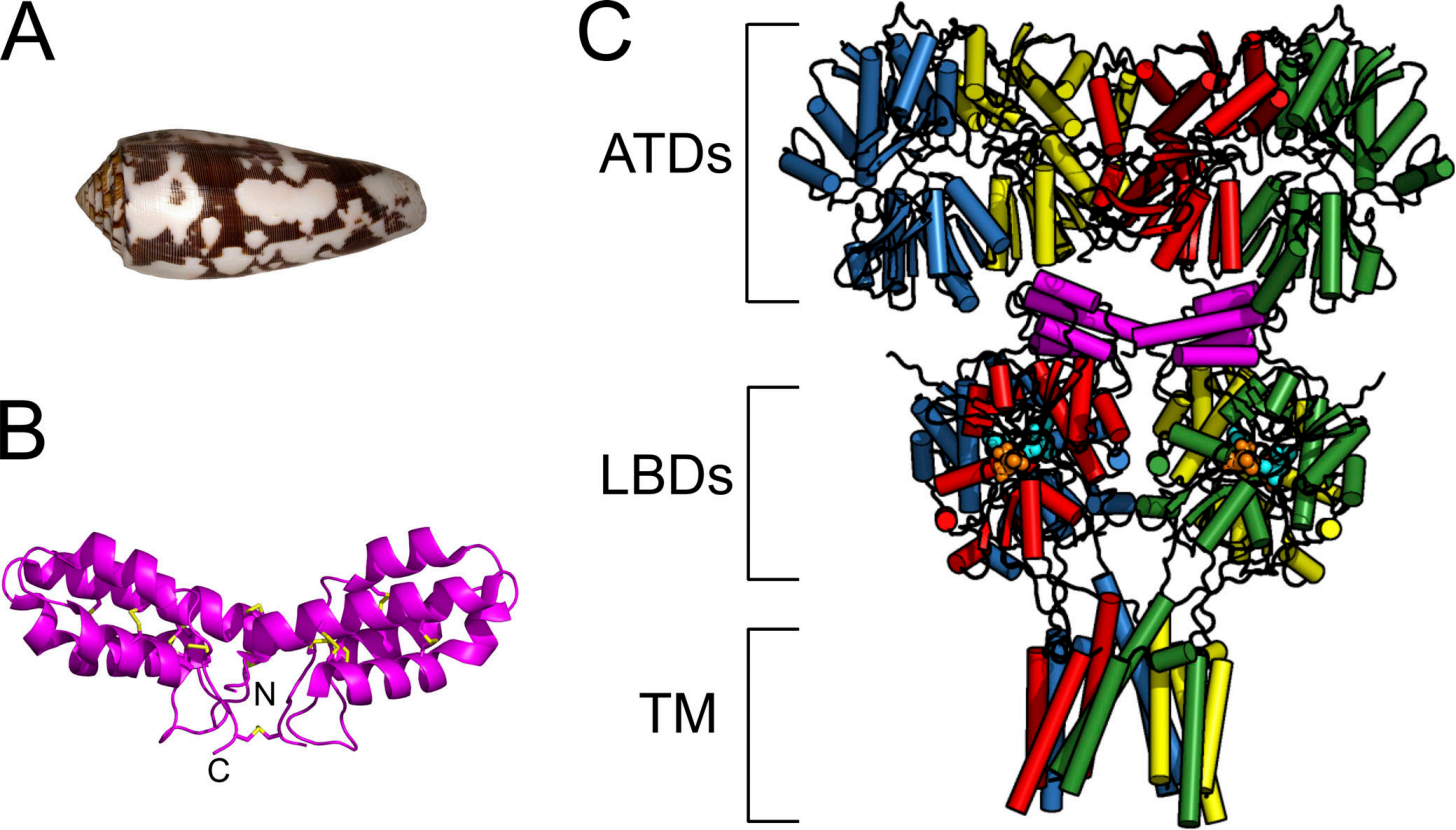
**Structure of CII toxin. (A)** Shell of a *Conus striatus* snail, the natural source of CII toxin. (urjsa, 2008 is marked with CC BY-SA 2.0). **(B)** Crystal structure of the toxin homodimer; each CII monomer is a four-helix bundle containing five disulfide bridges, connected to another monomer via additional three disulfide bridges (yellow; PDB accession no. 4U5H; Chen et al., 2014). **(C)** Crystal structure of the full-length AMPA receptor (subunit GluA2) in complex with a toxin (magenta), partial agonist kainate, and desensitization blocker (R, R)-2b (PDB accession no. 4U5D; Chen et al., 2014); each AMPA receptor subunit is colored differently with the domains indicated with square brackets as: ATDs, amino terminal domains; LBDs, ligand binding domains; and TM, transmembrane region. Red and blue subunits are forming one and green and yellow the other LBD dimer. The V-shaped toxin dimer (magenta) sits on top of LBDs.

The discovery of Con-ikot-ikot (CII) toxin added the AMPA (α-amino-3-hydroxyl-5-methyl-4-isoxazole-propionic acid) type glutamate receptors (AMPA receptors) to the repertoire of conotoxin targets (Walker et al., 2009). CII (from *Conus striatus*) is an unusual member of the conotoxin superfamily. Although it is rich in the typical disulfide linkages, its pattern is distinct from that in any existing conotoxin family, and it contains 86 versus the usual 20–30 amino acids (Fig. 1, A and B; Robinson and Norton, 2014).

AMPA receptors are the main mediators of excitatory neurotransmission in the vertebrate central nervous system. Their fast kinetics generally make them the first glutamate receptor subtype in the postsynaptic membrane to be activated by glutamate released from synaptic vesicles. Once bound by glutamate, their integral ion channel opens to allow Na^+^ influx, initiating membrane depolarization. The binding of glutamate, however, keeps the channel open for only a fraction of a millisecond because the receptor inactivates rapidly despite glutamate still being bound, a phenomenon known as desensitization. Desensitization plays a role in the normal synaptic transmission (Jones and Westbrook, 1996; Otis et al., 1996; Trussell et al., 1993) and appears important for the development of the nervous system (Christie et al., 2010).

AMPA receptors are tetrameric proteins composed of different combinations of subunits GluA1-A4. Each subunit is composed of four domains: amino terminal domain (ATD), ligand binding domain (LBD), transmembrane region with the ion channel, and cytoplasmic domain (Fig. 1 C; Sobolevsky et al., 2009). ATDs and LBDs form the extracellular part of the receptor, protruding into the synaptic cleft. ATDs are separated from LBDs by flexible linkers, leading to practically no interaction between the two domains and creating free space between them (Yelshanskaya et al., 2016). As shown by the crystal structure of an AMPA receptor in complex with the CII toxin, this cavity between the ATDs and LBDs in the tetramer is exactly where the toxin dimer binds, interacting mainly with the LBDs (Fig. 1 C; Chen et al., 2014). The V-shaped toxin dimer fits neatly on top of the AMPA receptor LBDs: four LBDs in the receptor are arranged as a dimer-of-dimers and each toxin monomer sits on top of one dimer. Thus, as long as the toxin is bound, the dimers remain intact. Intact active dimers negate the principal route to desensitization: the breaking of this interface within individual dimers (Sun et al., 2002). As a consequence, CII toxin blocks AMPA receptor desensitization, prolonging receptor activation and over-exciting the postsynaptic neuron (Walker et al., 2009). This mechanism is the basis of CII toxicity. Given the block of desensitization, it was somewhat surprising to see that crystal structures of an AMPA receptor in a complex with CII toxin, partial agonist, and another desensitization blocker resulted in a closed ion channel pore (Chen et al., 2014).

Here, we explore the mechanism of CII toxin action on AMPA receptor activity over hundreds of thousands of single-channel transitions. The toxin blocks desensitization of AMPA receptors effectively, achieving 50% desensitization block at the concentration of 5 nM (*EC*_50_). However, when compared to other desensitization blockers, such as cyclothiazide (CTZ) and (R, R)-2b, the toxin is a poor stabilizer of the fully open channel, leading to frequent and brief closures and an overall activation, considerably below the maximum attainable. We propose the reason for this inability to stabilize the active state of the receptor lies in the toxin’s unique mode of binding, distinct from other desensitization blockers. While CTZ and (R, R)-2b both bind within individual dimers, leaving them free to move with respect to each other, the toxin “locks” the two dimers at a fixed angle. Thus, the toxin fully immobilizes the LBD layer of the receptor, which appears incompatible with a stable fully open ion channel.

The presented comparison of the three desensitization blockers augments the existing evidence for the paradoxical observation that the block of desensitization in glutamate receptors is not necessarily synonymous with high activity. Our measurements of the *EC*_50_ of CII toxin, as well as its average residence time on the receptor, establish CII as a promising probe with potential applications for imaging experiments.

## Materials and methods

### Toxin expression and purification

**Figure 2.**
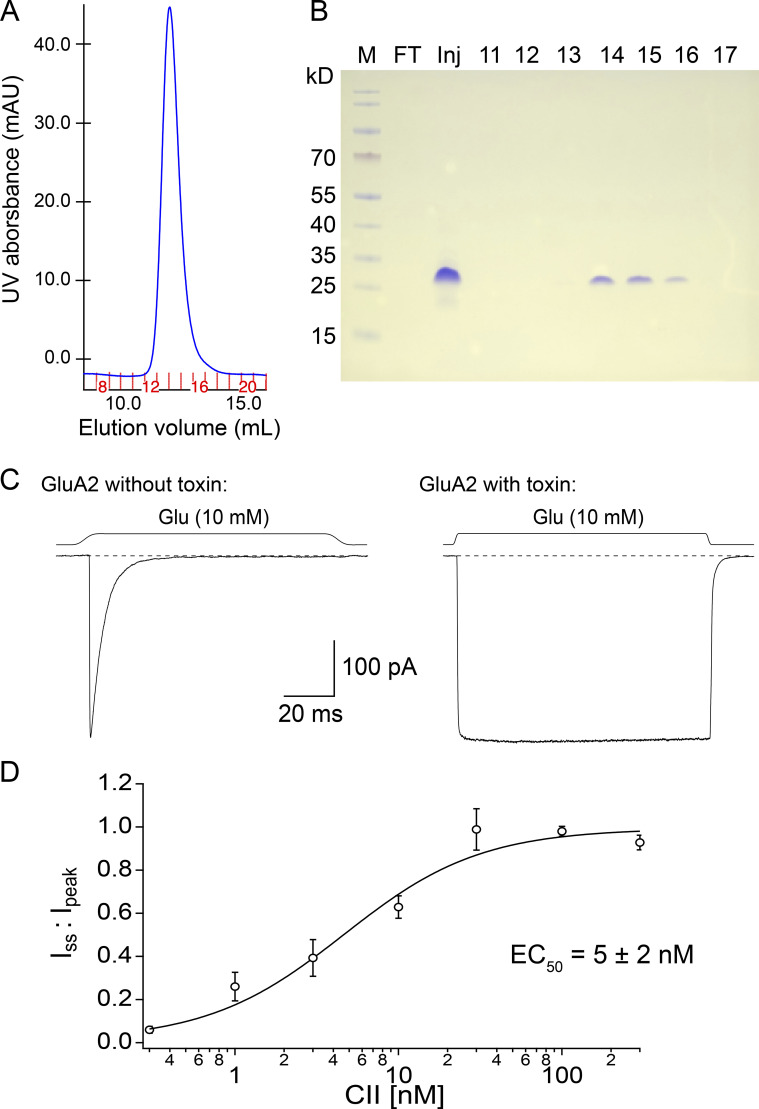
**CII toxin purification and characterization. (A)** Size-exclusion chromatogram (SEC) of purified CII toxin with fraction numbers indicated in red. **(B)** Coomassie-stained non-reducing SDS-PAGE gel of the SEC fractions. Wells marked 11–17 are SEC fractions, Inj is the concentrated sample injected onto the SEC column, and FT is the flow-through after concentrating the sample and before loading it onto the SEC column (to check for any protein loss). Fractions 14–16 contain the purified CII dimer. **(C)** The activity of the purified toxin was checked with outside-out patches: left trace shows the current produced by GluA2 AMPA receptors in the presence of glutamate (10 mM) without CII bound and right trace is from a different patch with CII toxin bound (after overnight incubation in 100 nM toxin). The dashed line indicates baseline and square trace above solution exchange in the respective patch. Voltage was approximately −40 mV in both recordings. **(D)** CII toxin dose-response curve for AMPA receptors (0.3–300 nM CII, *n* = 3–8 patches/toxin concentration). Toxin effect was measured as desensitization block, i.e., ratio of steady-state current over peak current for each patch (one for complete desensitization block). Fit by Hill equation gave *EC*_50_ of 5 ± 2 nM and Hill slope of 1 ± 0.3 (95% confidence interval).

The plasmid pET32b containing the mature sequence for the cone snail toxin CII (amino acids 38–123, UniprotKB accession no. P0CB20), preceded by a Trx fusion tag, a Strep tag, and a HRV 3C cleavage site was a kind gift from Eric Gouaux (Chen et al., 2014). The vector was transformed into Origami B (DE3) *Escherichia coli* cells and grown in LB medium until OD_600_ reached 0.8–1.0. The cultures were then induced with 100 µM IPTG at 16°C and harvested 16–20 h after induction. For cell lysis, the pellet was resuspended in lysis buffer (50 mM Tris-HCl, pH 8, 150 mM NaCl, 5 mM EDTA, 1 mM Pefablock, 50 µg/ml lysozyme, and 25 µg/ml DNaseI) and sonicated on ice for a total of 5 min. To collect the supernatant, the lysate was centrifuged at 20,000 rpm with a Fiberlite F21-8x50y rotor for 40 min and loaded onto a YMC ECO15/120V0V column packed with 10 ml Strep-Tactin Superflow high capacity resin. First, the column was washed extensively with Strep buffer A (20 mM Tris-HCl, pH 8, 150 mM NaCl, and 1 mM EDTA) and then eluted in one step with Strep-buffer B (20 mM Tris-HCl, pH 8, 150 mM NaCl, 1 mM EDTA, and 2.5 mM *d*-desthiobiotin). A 30-kD cut-off Amicon centrifugal filter was used to concentrate the eluted protein, and 3C protease (1:100) was added to cleave off the Trx tag overnight at 4°C. The next day, two volumes of methanol were added to the digested protein in order to precipitate the Trx tag, incubated at 37°C for 10 min, and centrifuged at 4,000 rpm for 10 min. The supernatant was recovered and concentrated using a 3-kD cut-off Amicon centrifugal filter, exchanging the buffer with consecutive concentration runs to 20 mM Tris-HCl, pH 8, 150 mM NaCl, and 1 mM EDTA. The resulting concentrate was left at room temperature for 24 h before the addition of 1 mM GSH and was further incubated at room temperature for another 24 h to promote dimer formation. The GSH-treated sample was diluted 10-fold with SP_A buffer (30 mM NaAc, pH 4.2) and loaded onto a HiTrap SP HP 1 ml prepacked ion exchange column. The protein was eluted using a gradient from 100 to 250 mM NaCl. Ion exchange fractions were run on SDS-PAGE to identify fractions containing toxin dimers. These fractions were concentrated using a 3-kD cut-off Amicon centrifugal filter and loaded onto a Superdex 75 10/300 column equilibrated in 10 mM HEPES, pH 7.5, and 150 mM NaCl (Fig. 2 A). Toxin dimer was identified by SDS-PAGE (Fig. 2 B), and the corresponding fractions were pooled and concentrated using a 3-kD cut-off Amicon centrifugal filter to a final concentration of 0.9 mg/ml. The toxin was stored at 4°C after the addition of 0.01% NaN_3_ until further use.

### Electrophysiology

All recordings were performed with the wild type GluA2 subunit of AMPA receptors in the flip form, unedited (Q) at the Q/R site in the channel pore. EGFP was present downstream from the GluA2 coding region after an IRES to mark the transfected cells. Wild type AMPA receptors were expressed transiently in HEK293 cells (RRID: CVCL_0045, ACC No. 305, obtained from Leibniz-Forschungsinstitut Deutsche Sammlung von Mikroorganismen und Zellkulturen GmbH, tested negative for mycoplasma) using calcium-phosphate precipitation or PEI method as described previously (Baranovic et al., 2016; Riva et al., 2017). Cells were maintained in MEM Eagle medium (PAN-Biotech GmbH) supplemented with 10% (vol/vol) FBS and antibiotics (penicillin [100 U/ml] and streptomycin [0.1 mg/ml]; PAN-Biotech GmbH). For macroscopic recordings, cells were recorded 48–72 h after transfection, and for single-channel recordings, 24 h after transfection. In both cases, we used the outside-out patch configuration.

Recording pipettes were pulled from borosilicate glass and had resistances (when filled with internal solution) of 5–10 MΩ for macroscopic recordings and 10–20 MΩ for single-channel recordings. The internal (pipette) solution in all cases contained (in mM) 115 NaCl, 1 MgCl_2_, 0.5 CaCl_2_, 10 NaF, 5 Na_4_BAPTA, 10 Na_2_ATP, and 5 HEPES, titrated to pH 7.3 with NaOH. The external solution consisted of (in mM) 150 NaCl, 0.1 MgCl_2_, 0.1 CaCl_2_, and 5 HEPES, titrated to pH 7.3 with NaOH. Desensitization blockers CTZ and (R, R)-2b were added to the external solution at the final concentration of 100 and 10 μM, respectively, where noted. Stock solutions of both drugs were prepared in DMSO (at 50 and 100 mM, respectively) and stored at −20°C. CTZ was obtained from Hello Bio, and (R, R)-2b was a kind gift from Eric Gouaux. Receptors were activated with an external solution containing 10 mM glutamate and 5 mM sucrose to visualize the flowing stream from the fast perfusion tool.

CII toxin was either present in the perfusing external solution at 500 nM when observing binding of the toxin or in the bath external solution at saturating concentrations (350 nM–45 µM) for measuring the unbinding rate and performing trace idealization, with or without additional desensitization blockers. For constructing the dose-response curve (Fig. 2 D), HEK293 cells expressing wild type GluA2 receptors were incubated in culture dishes overnight at various toxin concentrations. Overnight incubation ensured sufficient time for toxin binding to the receptors, resulting in the complete block of desensitization in saturating toxin and incomplete block in sub-saturating toxin concentrations (Fig. 2 C).

We applied glutamate (with and without toxin, CTZ or (R, R)-2b) to outside patches via perfusion tools made from custom-manufactured four-barrel glass (Vitrocom; Plested and Poulsen, 2021). The 10–90% solution exchange time was measured from junction potentials at the open tip of the patch pipette at the end of each experiment and it was found to be <300 μs. Patches were clamped at −40 to −60 mV for macroscopic records and at −80 mV for single-channel currents, unless stated otherwise. For single-channel recordings, we regularly applied gentle suction to deform the patch and reduce the number of active channels. Currents were filtered at 10 kHz (−3 dB cut-off, 8-pole Bessel, Axopatch 200B amplifier) and recorded using AxoGraph X (Axograph Scientific, v1.7.6) via an Instrutech ITC-18 interface (HEKA) at a 20-kHz sampling rate.

### Analysis of macroscopic recordings

Ad hoc analysis during experiments and pre-processing of data was done in AxoGraph. Traces were averaged, baseline-corrected, and selected for fitting of exponentials before export. For the toxin dose-response curve (Fig. 2 D), the ratio of steady-state current to the peak current (I_ss_/I_peak_) was determined for each patch at a range of toxin concentrations (0.3–300 nM, *n* = 3–8 patches per toxin concentration). Data were fit in Igor Pro (v7.08) with a Hill equation

$$\frac{I_{ss}}{I_{peak}}=b+\frac{m‐b}{1+{\left(\frac{\left[EC_{50}\right]}{\left[T_{x}\right]}\right)}^{n}},$$ (1)

where *b* is the baseline steady-state current without toxin (fixed to 0.01 for fit), *m* is the maximum block of desensitization, [*EC*_50_] is the midpoint concentration, *n* is the Hill slope, and [*T*_*x*_] is toxin concentration.

### Single-channel recordings

**Figure 3.**
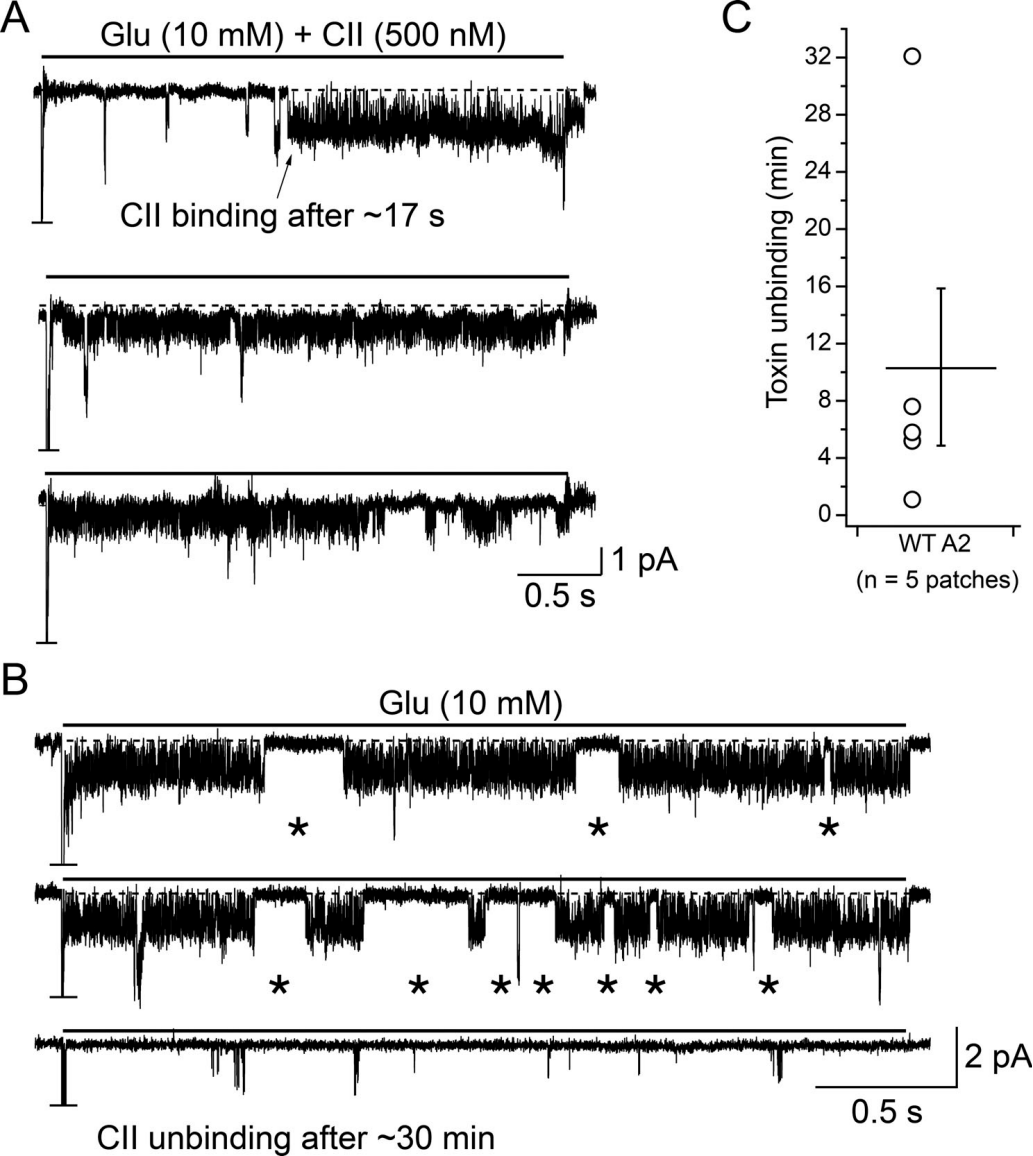
**Binding and unbinding of toxin to individual AMPA receptors. (A)** Current trace showing the binding of the toxin to one AMPA receptor in a patch containing multiple receptors, as indicated by the truncated peak current response at the start of glutamate application. Arrow indicates toxin binding event after about 17 s. A horizontal black line above the current trace indicates a jump from perfusing external solution (free of glutamate and toxin) into a perfusing solution containing glutamate (Glu) and the toxin (CII) for 3 s. Jumps were repeated as long as the patch was stable. **(B)** To detect toxin unbinding, patches with bound toxin were perfused in toxin-free solutions (3 s jumps into 10 mM Glu as described in A). In this example, long bursts of activity characteristic for toxin-bound AMPA receptors (top two traces) were replaced by short bursts of activity interspersed by long closures (bottom trace), after about 30 min. In both, A and B, the dashed line indicates the baseline. Voltage was clamped at −80 mV for both traces. Asterisks mark longer shut epochs (>5 ms) during the toxin-bound phase. **(C)** After binding of the toxin to one AMPA receptor in a patch, it took on average 10.4 ± 5.5 min (*n* = 5) to observe unbinding in toxin-free solutions, with regular 3 s jumps into glutamate.

For single-channel analysis, records were digital Gaussian-filtered (1–2 kHz) and selected for export with AxoGraph. The time needed for CII toxin to unbind from single GluA2 wild type receptors was determined by perfusing toxin-bound receptors in a toxin-free external solution (containing glutamate only) until toxin unbinding was observed as a sharp drop in the number of channel openings (Fig. 3 B).

Recordings were acquired in Axograph in an episodic manner (i.e., recording of each patch consisted of tens to hundreds of episodes with each episode containing one exposure of the patch to agonist). For each patch, amplitude points from the duration of agonist application (3 s) from each episode were concatenated into a single (continuous) current trace. An all-point-amplitude histogram was then generated for every patch in Igor Pro (v7.08; WaveMetrics), fit with a multi-peak Gaussian mixture function, and normalized for display. Fits from various patches belonging to the same condition (Glu + CTZ, Glu + CII, Glu + (R, R)-2b, or Glu + CII + (R, R)-2b) were plotted on the same graph to show variability across patches within a condition and to compare the four conditions.

Idealization of single-channel records was performed only on patches with a baseline root mean square noise of <300 fA (with 5 kHz Butterworth low-pass filter), in a PYTHON-based, open-source single-channel analysis application with a graphical user interface (ASCAM, http://www.github.com/AGPlested/ASCAM). ASCAM was optimized to handle multi-episode files and allow the user to select and mark traces for further analysis. Traces were saved as .MAT files (the MATLAB native format, for compatibility with other third-party software) and imported back into AxoGraph or Igor Pro for further analysis. Traces were filtered and the baseline was subtracted using a linear fit to the regions of baseline. Detection of open and closed channel events was limited to the interval over which agonist was applied by automatically thresholding the piezo command voltage. This procedure excluded any spurious detection from intervals of baseline. Due to desensitization block, in most patches, the channel continuously switched between briefly occupied open and closed states for as long as the agonist was present. We regularly observed four open levels for each active AMPA receptor. Idealization of single-channel records was done in ASCAM with a multiple threshold-crossing algorithm after filtering and baseline correction of the current traces. Each threshold was taken from the bisector of the adjacent open levels. For some patches, the threshold between the first open level and the zero baseline shut level was adjusted by hand to reduce spurious event detection from the baseline noise. These open levels were specified by the user from inspection of the all-point-amplitude histograms. For a typical idealization, we set the full amplitude to −2.4 pA and the sublevels equally spaced at 600 fA intervals. Dead time (duration of the shortest event that could be accurately detected) during idealization was set to 130 µs. We typically used interpolation (fivefold) to refine the determination of the threshold crossing time above this cut-off. The idealization process returned a numbered list of events (of channel opening times, levels, and the interspersed closed times). The list of events for each patch was exported as a text file for further analysis in Igor Pro.

Dwell time intervals (*t*) were log-transformed (Eq. 2; Sigworth and Sine, 1987),

$$x=\log\left(t\right),$$ (2)

The probability density function was appropriately transformed (Eq. 3),

$$f\left(x\right)=\frac{dP}{dx}=\frac{dP}{d\log\left(t\right)}=\frac{dt}{d\log\left(t\right)}\cdot \frac{dP}{dt}=2.3\cdot tf\left(t\right)\text{.}$$ (3)

Dwell time histograms were then fit with a single component function, or for two components, according to Eq. 4.

$$f\left(x\right)=2.3\left(\frac{a_{1}\cdot 10^{x}\cdot \exp\left(-\frac{10^{x}}{\tau_{1}}\right)}{\tau_{1}}+\frac{a_{2}\cdot 10^{x}\cdot \exp\left(-\frac{10^{x}}{\tau_{2}}\right)}{\tau_{2}}\right)\text{,}$$ (4)

where *τ*_1_ and *τ*_2_ are time constants of the exponential components of the fit (given in Table 2). The single-component function was equivalent to Eq. 4 with *a*_2_ fixed to zero. The goodness of fit was determined from the minimum value of χ^2^, the standard procedure in Igor Pro.

To determine the “fractional activity” of a single GluA2 receptor in complex with different desensitization blockers, we calculated the fraction of the maximum charge (*Q*_Frac_) transferred during the single-channel activity using Eq. 5:

$$Q_{Frac}=\frac{\sum_{n=1}^{4}O_{n}\cdot A_{n}}{A_{\max}},$$ (5)

where *O*_n_ is the fraction of time spent at each open level *n* (1–4), determined from the idealization, and *A*_n_ is the amplitude of that level; *A*_max_ is the amplitude of the maximum open level in the recording (that is, *A*_4_). The maximum charge is defined as the charge that would be transferred across the channel if the channel was open at *A*_max_ for the whole duration of agonist application.

Fractional occupancy (Fig. 6 and Table 1) was determined from the sum of dwell times in a particular state normalized to the total time of the idealized trace (during the glutamate jump). Normalized frequency of visits (Fig. 6 and Table 1) was calculated from the ratio of the number of events for each state divided by the total number of events detected in the patch. The number of events detected was between 5,000 and 60,000 per patch.

Multiple comparison tests were done with Tukey’s honestly significant difference (HSD) post hoc test (Igor Pro). Measurement of the probability of difference in component amplitudes was done with a randomization test (DC-Stats, http://www.github.com/aplested/dc-stats) that also provided the effect size and confidence intervals. The spread of the data is indicated as the standard deviation of the mean unless stated otherwise. Graphs were plotted and fitted in Igor Pro. Visualization of molecular structures was done in PyMOL (The PyMOL Molecular Graphics System, V1.8.4.0 Schrödinger, LLC).

## Results

### Purification of CII toxin

CII toxin was purified as described in Chen et al. (2014). Each individual purification started from 12 liters of *E. coli* cultures. Despite large starting volumes, the final toxin yield was modest, similar to previous reports (Chen et al., 2014). Of the three purifications, two resulted in ∼100 μg and one in ∼40 μg of CII toxin. The final product migrated as a dimer on a non-reducing SDS-PAGE gel (Fig. 2, A and B). The CII dimer is ∼20 kD in size, but migrates like a larger protein on SDS-PAGE gels.

The activity of each batch of the purified toxin was tested in macroscopic recordings, either by perfusing AMPA receptors in an outside-out patch with 500 nM toxin and 10 mM Glu or by incubating HEK cells expressing AMPA receptors in a saturating concentration of the toxin (≥350 nM; Fig. 2 D). AMPA receptor desensitization block was used as a measure of CII toxin activity, determined as the ratio of the steady-state current over the peak current. In a complete block of desensitization, the AMPA receptor current was sustained as long as glutamate was present, resulting in a square current response (Fig. 2 C, right).

To determine the *EC*_50_ value of the toxin, its ability to block AMPA receptor desensitization was tested over a range of different concentrations (0.3–300 nM) following a long incubation (12 h). The resulting dose-response curve was fit with a Hill equation (see Materials and methods) giving *EC*_50_ of 5 ± 2 nM and Hill slope of 1 ± 0.3 (95% confidence interval; Fig. 2 D).

### Binding and unbinding of CII toxin to individual AMPA receptors

We next sought to observe the binding and unbinding of CII toxin to individual AMPA receptors. To observe CII binding, AMPA receptors in a patch were perfused with 10 mM glutamate and 500 nM CII. In patches containing more than one AMPA receptor, binding of CII to only one receptor was regularly seen because nanomolar CII concentration led to a slow association rate (Fig. 3 A). This slow binding also meant that patches often had to be perfused for minutes before a binding event was observed. Toxin binding was evident as a sudden change from a largely quiescent, desensitized state, during the 10 mM glutamate pulse, to frequent, brief openings and closures, without long, closed periods (>100 ms), as expected from desensitized GluA2 receptors in saturating glutamate (Carbone and Plested, 2012; Fig. 3 A). Occasional longer closures (>5 ms), which could represent visits to desensitized states, remained (Fig. 3 B, and dwell time analysis below). Desensitized intervals in the 5–9 ms range may occur in the presence of LBD desensitization block due to conformational transitions of the LBD-transmembrane linker domains (LBD-TMDs; Zhang et al., 2017).

The majority of the closures (shuttings) we refer to in the presented single-channel records are shorter than 5 ms and indicate the transition of the ion channel from a conductive to a non-conductive state. We cannot say with certainty these are not short (<5 ms) desensitized intervals. All the recordings presented here were done in the presence of modulators that allow sustained activity (i.e., block desensitization), and the vast majority of these shuttings are of short duration (∼200–500 µs), and so most likely do not correspond to desensitization. In wild type channels without modulators, desensitization generates long time constants in the shut time distribution (>20 ms) that are eliminated by desensitization blockers such as cyclothiazide (Zhang et al., 2017), and according to our data, by (R, R)-2b and the CII toxin, too. As a rule of thumb, any such rapidly-attained desensitized states, if they were introduced by the toxin itself, should have been visible in the macroscopic currents as a relaxation following the jump into glutamate (Fig. 2 C), but these responses were routinely square (in saturating toxin) and showed no current decay in the presence of glutamate.

Once the toxin was bound (either via perfusion of CII or following incubation of the receptors in a saturating CII concentration), the patch was perfused in a solution containing 10 mM glutamate without any toxin to observe unbinding. An unbinding event was noted when the AMPA receptor activity changed from frequent and brief openings and closures to long quiescent periods with rare bursts of activity (Fig. 3 B), corresponding to the activity of the unbound form. In agreement with the toxin’s nanomolar *EC*_50_, unbinding of the toxin took 10.4 ± 5.5 min (*n* = 5 patches; Fig. 3 C).

After establishing that the binding of the toxin to AMPA receptors blocks desensitization, while at the same time resulting in frequent switching between brief open and closed states, we next investigated how this behavior compares to other desensitization blockers.

### Comparison of CII toxin to other desensitization blockers

Upon establishing that purified toxin was active and that it blocked AMPA receptor desensitization at single-channel level, we next sought to compare CII toxin to two other desensitization blockers: cyclothiazide (CTZ) and (R, R)-2b. CTZ is the most widely used AMPA receptor desensitization blocker (Partin et al., 1994). Two molecules of CTZ bind within one AMPA receptor LBD dimer, holding it together and preventing desensitization (Sun et al., 2002). At a single-channel level, CTZ has been reported to increase open probability and burst duration, as well as decrease the occupancy of the lowest conductance level and increase the occupancy of higher conductance levels for both GluA1 and GluA4 homomers (Fucile et al., 2006; Zhang et al., 2017). (R, R)-2b targets the same binding sites as CTZ, spanning both sites in a dimer with a double-headed arrangement, but is less characterized and used than CTZ (Kaae et al., 2007). A single molecule of (R, R)-2b binds per one AMPA receptor LBD dimer with *EC*_50_ of 0.44 μM, an order of magnitude lower value than the *EC*_50_ of CTZ (5 μM). We tested (R, R)-2b here as it has been used in several AMPA receptor structures both with (Chen et al., 2014) and without the toxin (Dürr et al., 2014).

**Figure 4.**
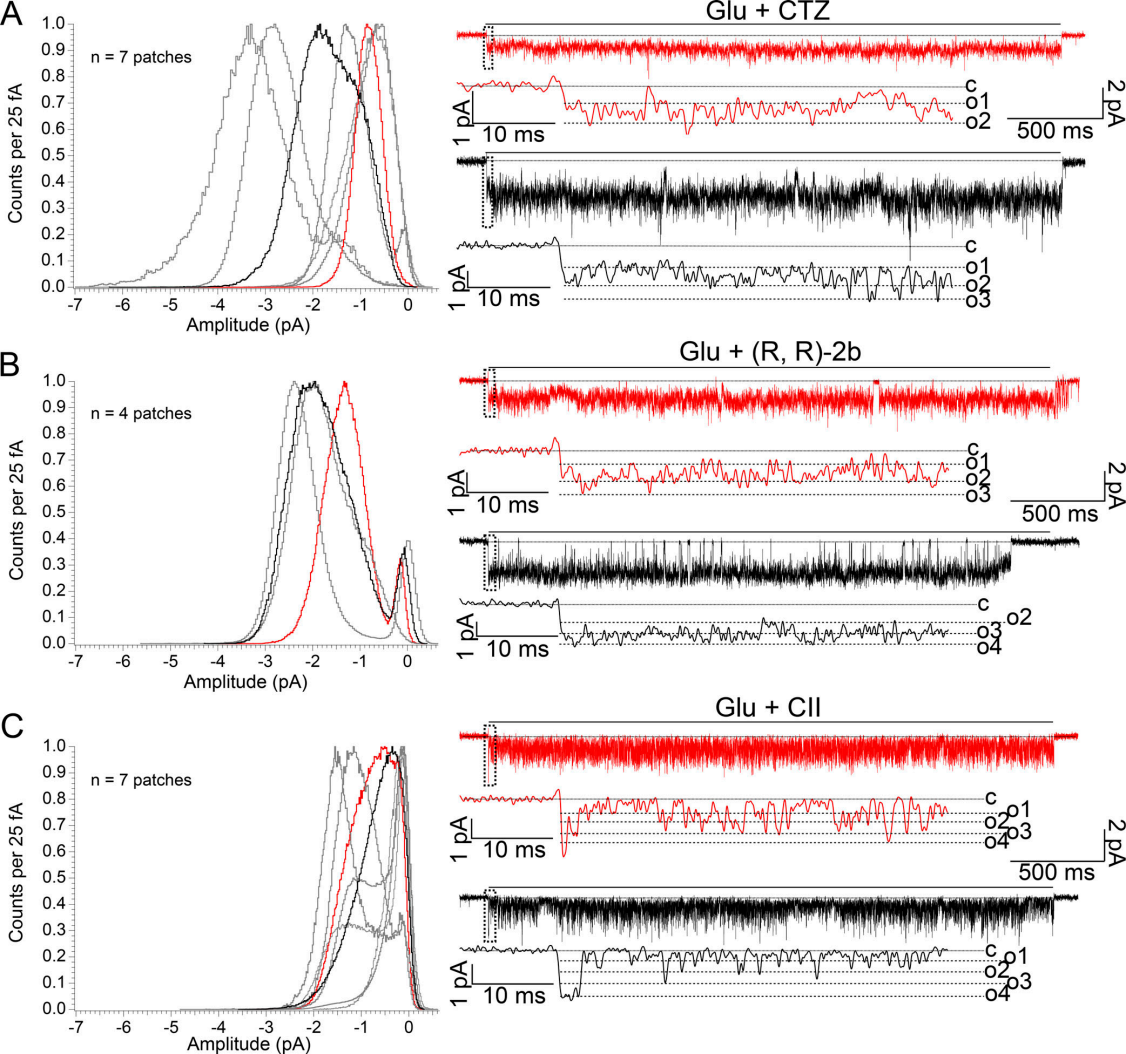
**Comparison of CII toxin with AMPA receptor desensitization blockers CTZ and (R, R)-2b. (A)** All-point-amplitude histograms of single-channel recordings of GluA2 wild type receptors perfused in glutamate (10 mM) and positive modulator cyclothiazide (CTZ, 100 μM), *n*_CTZ_ = 7 (∼56 s of recording in total). A histogram was made for each patch, using only the parts of the recording where the patch was perfused in glutamate (Glu) and CTZ. Histograms were normalized to the maximum value. Representative single-channel currents from two color-coded patches are drawn next to the corresponding histograms. Dashed box indicates the part of the trace shown at greater magnification below. Dotted lines are baseline (closed level [c] in zoom), and dashed lines are open levels in each patch (O1–O4). All openings are downwards, traces were obtained at approximately −80 mV and low pass filtered at 1 kHz for presentation. **(B and C)** Same as in A, but with different modulators: (R, R)-2b (10 μM; *n*_RR_ = 4; ∼81 s of recording in total), CII toxin (100–500 nM; *n*_CII_ = 7; ∼77 s of recording in total).

To compare the effects of CTZ, (R, R)-2b, and CII toxin on AMPA receptor single-channel activity, we generated all-point-amplitude histograms from recordings of individual GluA2 receptors made in each of the three conditions (Fig. 4). The histograms were made from parts of the recordings where the receptor was perfused with glutamate and the corresponding modulator, so any closed periods (the baseline around 0 pA) are a consequence of the receptor inactivity during agonist application (see also Materials and methods). The histograms show there is some heterogeneity in the channel activity between patches for each positive modulator. This was particularly the case in CTZ, where three out of seven patches showed pronounced peaks in the histogram around 0 pA, indicating the receptor spent considerable amounts of time in non-conducting states, whereas two patches exhibited high amplitudes over −2.4 pA at −80 mV. Although we cannot exclude the possibility there is more than one channel opening in these high-amplitude patches in CTZ, we did not observe any characteristics of double (or multiple) openings, which would be highly likely to occur in non-desensitizing conditions and high activity during 10 s of recording. In contrast, modulation by (R, R)-2b enabled the GluA2 receptor to open consistently to amplitudes over −2 pA at −80 mV, which we took to be the full level of activation. In this context, CII toxin was revealed as the poorest promoter of the full open state of AMPA receptors. When complexed with the CII toxin, the receptor frequently visited non-conductive states, and the lowest amplitude sublevels in the presence of 10 mM glutamate in all obtained patches. At the same time, visits to open amplitudes above −2 pA at −80 mV were less frequently observed than in CTZ. Particularly in (R, R)-2b, visits to the full-open state were dominant compared to the visits to the baseline, as indicated by small peaks around 0 pA for all patches. Overall, these recordings revealed that variability in the recordings (which we assess greatest in CTZ and least in CII) was apparently positively correlated with *EC*_50_ values—the lower the value, the lesser the variation.

Both CTZ and (R, R)-2b bind within the intra-dimer LBD interface. In principle, toxin and (R, R)-2b should not occlude each other’s binding, as demonstrated by complexes captured in crystal structures, together with partial agonists (Chen et al., 2014). To test whether immobilization of the LBD layer by toxin leads to an overall decrease in the activity of AMPA receptors, even in the presence of other desensitization blockers, we next sought to record the activity of single AMPA receptors in the presence of both toxin and (R, R)-2b.

### (R, R)-2b cannot overcome the low AMPA receptor activity induced by toxin binding

To investigate whether immobilization of the LBD layer by toxin is essential for its ability to reduce AMPA receptor activity, we recorded currents through single AMPA receptors treated with both toxin and (R, R)-2b. If locking the two LBD dimers with respect to each other by the toxin is indeed preventing AMPA receptors from stably occupying the most open level, then the presence of the toxin will have a negative impact on AMPA receptor activity, even in the presence of a potent positive modulator, such as (R, R)-2b. The challenging aspect of these recordings was to know that both modulators were bound to the receptor. In the majority of the recordings, the coverslip with HEK293 cells expressing wild type GluA2 receptors was incubated in a solution containing saturated toxin concentration. Almost all the patches from such cells contained receptors bound by the toxin, as evident from the characteristic single-channel activity induced by the bound toxin (Fig. 3). Patches with bound toxin were then perfused in saturating glutamate (10 mM) and (R, R)-2b (10 μM) for at least 10 s. Due to its sub-micromolar *EC*_50_ value (∼0.4 μM) and saturating concentration, (R, R)-2b was able to bind to AMPA receptors almost instantaneously if the sites were accessible. The reverse experiment, where (R, R)-2b was first allowed to bind to the receptors and subsequently toxin was added could not be performed satisfactorily. Patch recordings are unreliable and perfusing patches that did not give single-channel recordings consumed prohibitive amounts of the toxin preparation. Crystal structures of AMPA receptors in complex with the toxin, (R, R)-2b, and partial agonists (kainic acid or fluorowillardiine), where the receptors were first incubated in CII toxin (at 1:1.5 M ratio of receptor to toxin, respectively) and then (R, R)-2b, suggest that (R, R)-2b can bind to toxin-bound AMPA receptors (Chen et al., 2014), although the timescale may be longer. Crystal structures obtained with kainic acid and fluorowillardiine differed slightly, but the toxin binding pose was identical between the two. The binding of glutamate is not expected to promote a grossly distinct arrangement. However, in these co-crystal structures, it is not fully clear whether the toxin can stably hold the active dimer pose of each pair of LBDs in the absence of (R, R)-2b.

**Figure 5.**
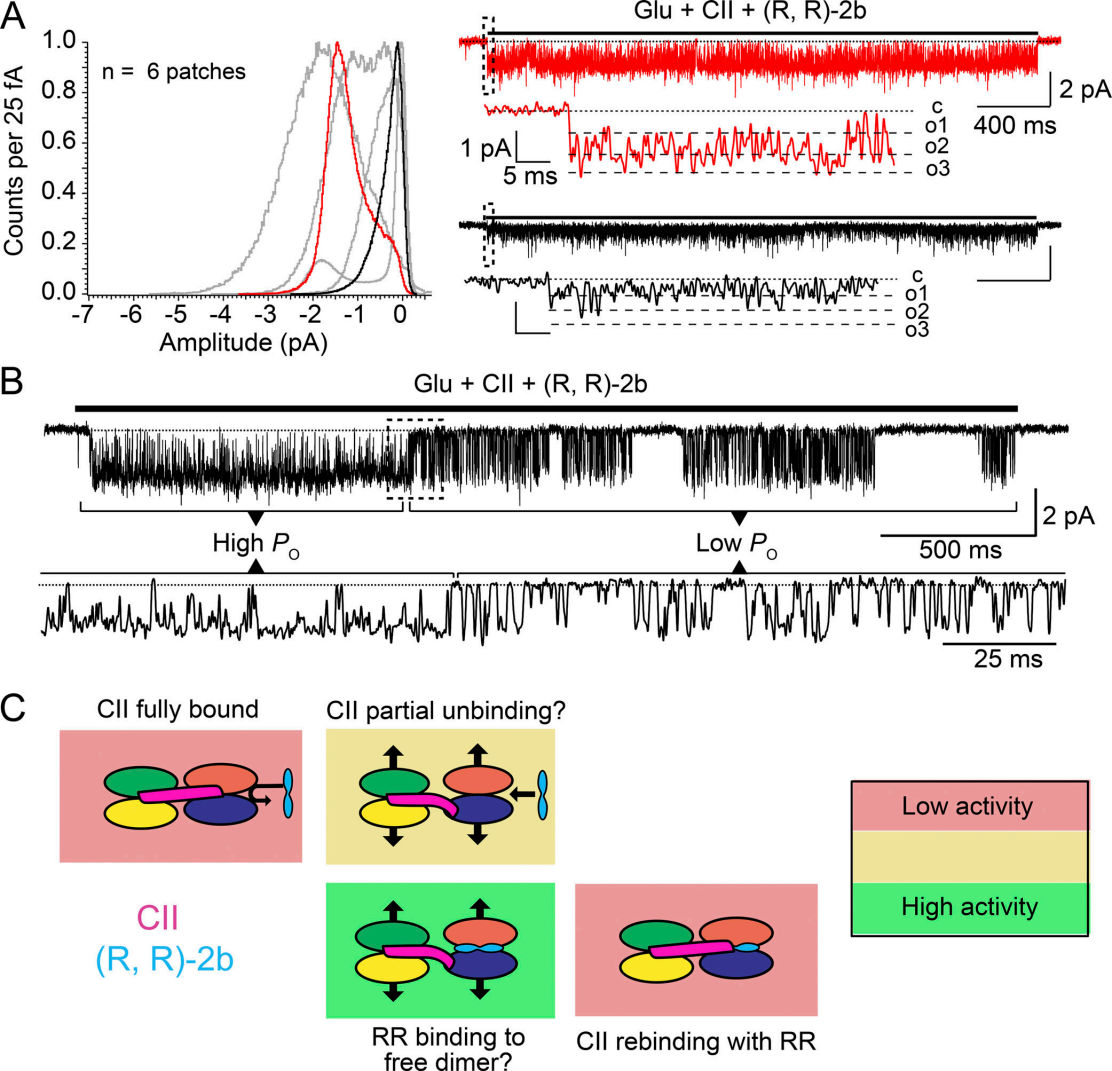
**AMPA receptor activity in CII toxin and (R, R)-2b. (A)** As in Fig. 4, a normalized histogram was made for each patch. Representative single-channel currents from two color-coded patches are drawn on the same scale. The red trace had comparatively high activity, whereas the black record shows much less activity. Dashed box indicates the part of the trace shown at greater magnification below (again with common scale). Dotted lines are baseline (closed level [c] in zoom), and dashed lines are open levels in each patch (O1–O3, O4 not reached). All openings are downwards, traces were obtained at approximately −80 mV and low pass filtered at 1 kHz for presentation. Patches were taken from cells incubated in 100–500 nM CII and exposed to 10 μM (R, R)-2b; *n*_RR+CII_ = 6, ∼72 sec of recording in total. **(B)** Example of a mode switch from high P_open_ activity (similar to (R, R)-2b alone and red trace from A) to low P_open_ activity (similar to CII alone and black trace from A) during a 3 s recording in the presence of 10 mM glutamate, 10 μM (R, R)-2b, and saturating CII. **(C)** Diagram indicating how binding and unbinding of CII toxin (magenta) and (R, R)-2b (cyan) might lead to different activity levels of an AMPA receptor (red sections, low activity; yellow sections, intermediate activity; green sections, high activity). LBDs of AMPA receptors (top view) are indicated by four differently coloured ellipses. Thick, black arrows indicate freedom of movement for LBDs.

All-point-amplitude histograms for receptors treated with toxin and (R, R)-2b were again heterogenous (Fig. 5 A), but showed more similarity to those of toxin-bound receptors than (R, R)-2b-bound receptors. Some records were of high activity (with frequent high conductances), but most had a lower activity with very rare high conductances, reaching amplitudes over −2 pA at −80 mV (Fig. 5 A). Critically, we observed switching between these two modes of activity, occasionally even within the same recording (with longer interstitial shut times; Fig. 5 B), indicating that the modes might come from modulator behavior and not receptors with different static properties. In Fig. 5 C, we outline a scheme of how such a mode switch could be due to the initial occlusion of the (R, R)-2b binding site by a fully bound toxin macromolecule. In this interpretation, the seconds-to-minutes dynamics of the toxin binding–unbinding reaction across multiple subunits determine the gating modes (see Discussion). However, a set of gating modes produced by simultaneous binding by CII and (R, R)-2b cannot be excluded.

In all-point-amplitude histograms, the area under the curve is proportional to the total time spent at a given amplitude, but there is no indication of the duration or frequency of visits that the channel makes to openings of specific amplitudes. In other words, these histograms do not distinguish between frequent and short openings versus rare and long openings to a specific amplitude. To gain more insight into the gating of receptors bound with the three desensitization blockers, we idealized single-channel traces to determine occupancy of closed and open states. We also reasoned that we should see differences in the dwell time distributions if the toxin and the modulator (R, R)-2b could both bind.

### Occupancy, frequency, and activity in idealized single-channel records

**Figure 6.**
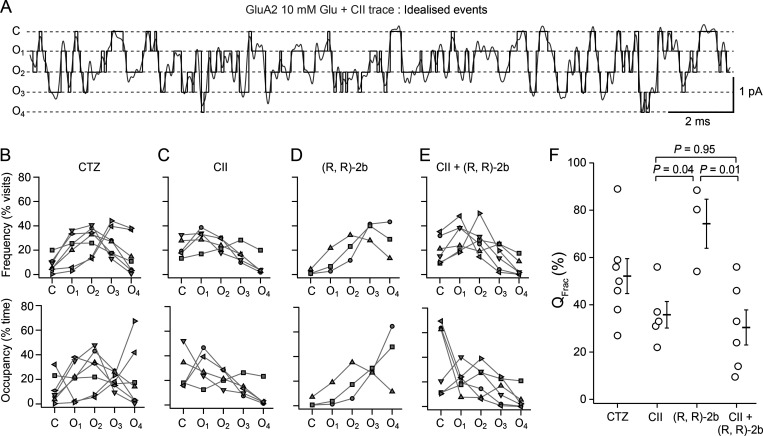
**Occupancy of closed and open levels for GluA2 bound by modulators of desensitization. (A)** An example of the idealization from ASCAM (black), overlaid on the current trace (grey; low-pass filtered at 1 kHz for presentation) obtained in glutamate (Glu, 10 mM) and toxin (CII, 500 nM). Dashed lines indicate the four open conductance levels (O1–O4) and the closed level, C. **(B–E)** CTZ (100 μM; B), toxin alone (100–500 nM; C), (R, R)-2b (10 μM; D) and toxin + (R, R)-2b conditions (E). The frequency of visits to each level (upper row) and occupancy of each level as a fraction of the total time (bottom row) are shown for each patch. Different symbols in each condition represent different patches. **(F)** Fractional activity (*Q*_Frac_, %) was determined as the ratio of the transferred charge to the charge that would be transferred if the channel were open continuously to the maximum open amplitude; CTZ: 53 ± 7% (*n* = 7), CII: 36 ± 6% (*n* = 5), (R, R)-2b: 74 ± 10% (*n* = 3), and toxin and (R, R)-2b: 30 ± 6% (*n* = 6). Horizontal bars are mean values and bars represent standard error of the mean. Probabilities of no difference shown are from Tukey’s HSD two-tailed test.

We idealized single-channel records with the threshold crossing method (see Materials and methods), using multiple thresholds between user-defined open levels (Fig. 6). As summarized in Table 1, four similar open levels were identified for receptors bound by either CTZ, toxin, (R, R)-2b, or exposed to both CII and (R, R)-2b. These data are quite similar to those reported previously for GluA2 with CTZ bound (Prieto and Wollmuth, 2010). With only toxin bound to its LBDs, the receptor spent the least fraction of time (6 ± 4%) at the highest amplitude level, open level 4, indicating rare and short-lived visits to the highest amplitude state (Table 1, “fraction occupied”). The receptor spent similar fractions of time across the closed states and the other three open sublevels (C: 27 ± 7%; O1: 30 ± 6%, O2: 22 ± 3%; O3: 14 ± 3%; Fig. 6, B–E, bottom). For (R, R)-2b, the occupancy profile of the receptor changed, with the biggest differences at the closed, O1, and O4 (most open level). The receptor now spent almost 10-fold less time in closed states compared to the CII-bound receptor (in (R, R)-2b the closed state occupancy was 3 ± 2%; P of no difference to CII was 0.053 by Tukey’s HSD multiple comparison test). Occupancy of the lowest open level (O1) was approximately three times lower (CII: 30 ± 6%, (R, R)-2b: 8 ± 6%), whereas the fraction of time spent at level 4 (O4) was approximately seven times greater (CII: 6 ± 4%, (R, R)-2b: 41 ± 16%; Fig. 6, C and D, bottom). For AMPA receptors exposed to both toxin and (R, R)-2b, the occupancies were very similar to toxin-only bound receptors, with the receptor spending 40 ± 12% of the time in the closed state, 22 ± 4% of the time at the first open level (O1), and 5 ± 3% of the time at the most open level (Fig. 6 E). The occupancy of the open levels O2 and O3 was not much different in toxin or (R, R)-2b, in each case, each level being occupied for ∼20% of the time (Fig. 6, B–E). Multiple comparisons for individual state occupancies across the four conditions did not yield probabilities of no difference that allowed us to exclude the null hypothesis at the 5% level. With the main variation occurring between closed and fully-open state occupancies, we examined the properties of the difference in these occupancies across conditions. For example, the average difference in occupancy (O4–C) for (R, R)-2b was 41–3% = 38 ± 18% (*n* = 3), whereas for CII the same difference was −21 ± 12% (*n* = 5). The probabilities of no difference between the CII and (R, R)-2b, or CII + (R, R)-2b and (R, R)-2b conditions were 0.045 and 0.009, respectively (Tukey’s HSD test). The differential occupancies (O4–C) between other conditions had a probability of no difference greater than 0.05.

**Table 1.** Amplitudes, frequencies, and occupancies of closed and open levels

Event amplitudes, frequencies, and occupancies	CTZ *n* = 7	CII *n* = 5	(R, R)-2b *n* = 3	CII + (R, R)-2b *n* = 6
Closed				
Amplitude (pA)	—	—	—	—
Frequency of visit	8 ± 2%	22 ± 4%	2 ± 1%	20 ± 5%
Fraction occupied	12 ± 4%	27 ± 7%	3 ± 2%	40 ± 12%
Open 1				
Amplitude (pA)	−0.67 ± 0.04	−0.59 ± 0.01	−0.64 ± 0.06	−0.64 ± 0.04
Frequency of visit	21 ± 5%	30 ± 4%	10 ± 6%	31 ± 5%
Fraction occupied	20 ± 5%	30 ± 6%	8 ± 6%	22 ± 4%
Open 2				
Amplitude (pA)	−1.3 ± 0.1	−1.2 ± 0.02	−1.3 ± 0.1	−1.3 ± 0.1
Frequency of visit	28 ± 4%	25 ± 2%	22 ± 6%	28 ± 5%
Fraction occupied	27 ± 6%	22 ± 3%	20 ± 9%	20 ± 5%
Open 3				
Amplitude (pA)	−2.0 ± 0.1	−1.75 ± 0.04	−1.9 ± 0.2	−1.9 ± 0.1
Frequency of visit	27 ± 4%	17 ± 3%	37 ± 4%	15 ± 4%
Fraction occupied	19 ± 2%	14 ± 3%	28 ± 1%	13 ± 4%
Open 4				
Amplitude (pA)	−2.7 ± 0.2	−2.3 ± 0.1	−2.7 ± 0.2	−2.6 ± 0.2
Frequency of visit	16 ± 6%	6 ± 3%	29 ± 9%	5 ± 3%
Fraction occupied	21 ± 9%	6 ± 4%	41 ± 16%	5 ± 3%

Idealization of single-channel recordings of GluA2 wild type receptors in glutamate (10 mM) and CTZ (100 μM), CII toxin (100–500 nM), (R, R)-2b (10 μM), or CII + (R, R)-2b, resulted in four open levels for each condition (Open 1–4). Open level amplitudes did not depend on the type of positive allosteric modulator used. Frequencies of visiting each level are expressed as a percentage of the total events and occupancies are expressed as a percentage of the total time the channel spent at a specific level. Number of patches for each condition (*n*) is indicated.

We also analyzed the relative frequencies at which each sublevel and the closed level were visited. The most prominent variations across the conditions were in the frequencies of visits to closed states. The closed level was visited about 10-fold more frequently in CII compared to (R, R)-2b (Table 1, “frequency of visit”; 22 ± 4% of all sojourns versus 2 ± 1%, respectively; P of no difference by Tukey’s HSD test: 0.015) and about 2.5-fold more frequently compared to CTZ (8 ± 2%; P of no difference: 0.036). There was a similarly small probability of no difference between the CII + (R, R)-2b condition compared with (R, R)-2b alone (0.02). For other states and across other conditions, we could not reject the null hypothesis at the 5% level. Due to the apparently large differences in frequency of visits to closed and fully open levels across the different conditions, we again took the differences of these frequencies (keeping their sign) and subjected them to the Tukey’s HSD multiple comparison test. Consistent with our observations for the occupancies, we found that the differential frequency between the closed and fully open state visits allowed us to reject the null hypothesis at the 0.05 level for two of the six pairwise comparisons. For (R, R)-2b alone, 27 ± 10% more of the total visits were to O4 compared to C, whereas for CII, 16 ± 6% of the total visits were to C versus O4 (P = 0.017 versus (R, R)-2b alone) and for CII + (R, R)-2b, 15 ± 7% of the total visits were to C versus O4, (P = 0.015 versus (R, R)-2b alone). For other comparisons, we could not reject the null hypothesis at the 5% level. This analysis is consistent with CII being rather poor at preventing the channel closing and dominating in the presence of (R, R)-2b, as the recordings suggested from simple inspection.

Single-channel activity is often expressed as open probability–the fraction of time in open states compared to the overall time. Since GluA2 shows four evenly-spaced sublevels when desensitization is blocked, this measure (which weights all openings equally independent of their amplitude) is somewhat inadequate, and in particular failed to distinguish between the high and low activity we observed in different conditions. To better assess the activity of the channel in each condition, we determined the fraction of maximum charge (*Q*_Frac_) passed by the channel as described in the Materials and methods section. In CTZ, the channel transferred 53 ± 7% (*n* = 7) of the maximum charge and in CII toxin, 36 ± 6% (*n* = 5). The channel was twice as active in (R, R)-2b (74 ± 10%, *n* = 3), with a probability of no difference between CII and (R, R)-2b of 0.04 (Tukey’s HSD test of *Q*_Frac_ across all four conditions). The presence of both CII and (R, R)-2b resulted in similarly low activity to CII alone (30 ± 7%, *n* = 6; P of no difference to CII alone was 0.95) with a wider range of values (Fig. 6 F). Unsurprisingly, the probability of no difference between the (R, R)-2b and CII + (R, R)-2b conditions was also low, at 0.01. These results are congruent with the frequency and occupancy analyses, with the conditions of both CII + (R, R)-2b and CII alone being very different from (R, R)-2b alone. The differences were greatest between the closed and fully open state occupancies, which also have the greatest effects on *Q*_Frac_.

### Single-channel dwell times with desensitization blockers

**Figure 7.**
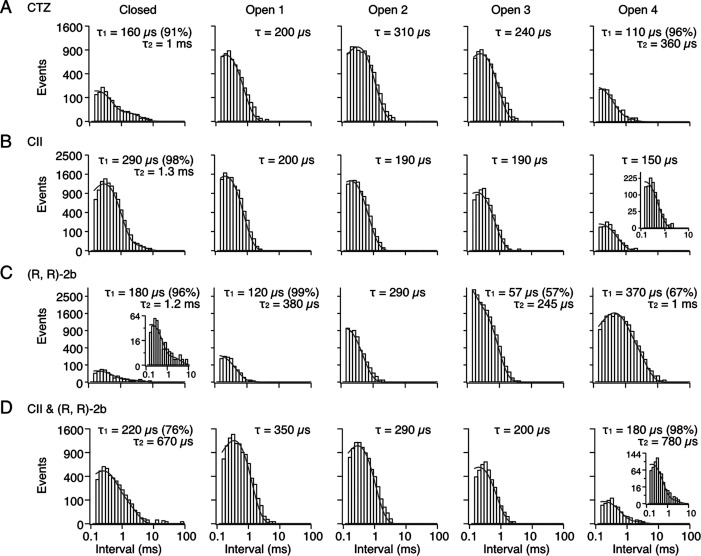
**Dwell times of AMPA receptors in the presence of desensitization modulators. (A–D)** Dwell time histograms were generated for the closed and each of the open levels (1–4), for CTZ (100 µM; A), CII toxin (100–500 nM; B), (R, R)-2b (10 μM; C), and toxin and (R, R)-2b (D) conditions. Shown are histograms from one representative patch for each condition. Each histogram was plotted on a log-square root scale and fit with one or two exponential components (grey curves). Mean time constants and amplitudes for each condition are given in Table 2.

To understand the effects on receptor activation more clearly, and particularly to distinguish between the gating of CII-bound receptors in the presence and absence of (R, R)-2b, we next generated dwell-time histograms for each condition (Fig. 7). All histograms were fitted with one or two exponential components, the values of which are summarized in Table 2. The duration of the first three open levels are similar across the four conditions (in the 150–300 µs range), and the presence of CTZ, CII toxin, (R, R)-2b or CII toxin + (R, R)-2b did not change them. The modulators also did not change the duration of visits to closed states. For each condition, the shut time distribution had two components, revealing at least two closed states in the presence of each modulator. These observations further support the idea that the modulators mainly exert their differential effects on activity by shifting the frequency of visits to the closed state and the fourth (largest amplitude) open level. The histogram fits indicated that there are at least two fully open (O4) states for each modulator, except CII alone, for which there was only one. Particularly, in five out of six recordings made with CII and (R, R)-2b present, a small second component (2.5 ± 1.8 ms, relative amplitude 2 ± 1%) was identifiable in the fully open state (O4). We excluded one patch from this condition because the O4 histogram was ill-defined with fewer than 20 events. This slow component was clearly absent in records from CII alone. The effect size (Hedges’ corrected d) was substantial (0.98; 95% confidence interval from bootstrap calculation was 0.6–2.3) and P of no difference to the CII + (R, R)-2b condition was 0.046 by the two-tailed randomization test (*n* = 5 patches). This small slow component represents one of the few detectable indications that (R, R)-2b could bind to receptors with toxin pre-exposure.

**Table 2.** Time constants from exponential fits to dwell time histograms

Fitted dwell time components (τ) in μs	CTZ *n* = 7	CII *n* = 5	(R, R)-2b *n* = 3	CII + (R, R)-2b *n* = 6
Closed				
τ_1_	300 ± 90	260 ± 50	170 ± 20	540 ± 210
τ_2_	2,400 ± 1,400	1,500 ± 300	1,080 ± 70	5,800 ± 4,200 (5)
*A*_1_	89 ± 7%	95 ± 2%	91 ± 8%	89 ± 6%
Open 1				
τ_1_	240 ± 20	240 ± 40	140 ± 20	180 ± 50
τ_2_	600 ± 300 (2)	—	400 ± 50	480 ± 320 (2)
*A*_1_	92 ± 6%	100%	95 ± 5%	93 ± 8%
Open 2				
τ_1_	280 ± 30	200 ± 10	220 ± 60	230 ± 40
τ_2_	570 (1)	—	—	400 ± 140 (3)
*A*_1_	97 ± 3%	100%	100%	86 ± 13%
Open 3				
τ_1_	260 ± 20	170 ± 20	180 ± 80	220 ± 50
τ_2_	700 (1)	330 ± 30 (3)	240 (1)	—
*A*_1_	99 ± 1%	85 ± 14%	86 ± 17%	100%
Open 4				
τ_1_	320 ± 70	180 ± 40	250 ± 80	270 ± 50 (5)
τ_2_	1,200 ± 400 (6)	—	900 ± 300	2,500 ± 1,800 (4)
*A*_1_	87 ± 4%	100%	75 ± 11%	98 ± 1%

Single-channel recordings of GluA2 wild type receptors were obtained in glutamate (10 mM) and the following positive allosteric modulators: CTZ (100 µM), CII toxin (100–500 nM), (R, R)-2b (10 μM), or CII + (R, R)-2b. Histograms of dwell times of closed and open levels (1–4) were obtained from idealized single-channel traces and fit with one or two exponential components (Fig. 7; *τ*_1_ and *τ*_2_). The amplitude of the first component (A_1_) is expressed in percent where appropriate, and *n* is the number of patches. Where only a subset of patches in the group could be fit, the number of patches in this subset used to obtain the time constant is given in parentheses.

## Discussion

The rich palette of positive modulators of AMPA receptors spans a range of chemical complexity from ions (Dawe et al., 2016; Partin et al., 1996) to small molecules (Vyklicky et al., 1991; Yamada and Tang, 1993), with CII toxin being the largest known. All act on the LBD layer, and have been largely characterized by macroscopic measurements of AMPA receptor currents. In such measurements, modulator activity is defined only relative to the original response or other modulators. Many AMPA receptor positive modulators are highly lipophilic (e.g., cyclothiazide), making washout difficult and implying that relative measurements are confounded by the loss of activity. Single-channel measurements, in contrast, allow an absolute definition of activity, and also give insight into the mechanism. Without auxiliary proteins, GluA2 has an activity (expressed as steady-state current) of about 3% in saturating glutamate (Carbone and Plested, 2012). As a measure of activity that also takes into account amplitudes occupied by the open channel, we determined the fraction of maximum charge (*Q*_Frac_) passed by the channel in single-channel records. Our measurements show that the addition of purified CII toxin increases AMPA receptor activity, and that the increase is limited (only about 12-fold, about 36% of the maximum), less than that produced by other small-molecule blockers of desensitization (25-fold increase to about 74% of maximal activity for (R, R)-2b). When the receptors are exposed to both, toxin and (R, R)-2b, the increase in activity is about 10-fold, to about 30%. This finding explains, at least in part, why toxin-bound crystal structures with partial agonists and (R, R)-2b had closed ion channel pores (Chen et al., 2014). In these conditions, the energy landscape remains tilted in the direction of inactive states.

Although the toxin blocked desensitization, prolonged shut periods characteristic of desensitized receptors were occasionally seen. These closures could be desensitization events with the toxin fully bound, although partial toxin unbinding (from some, but not all subunits), followed by reassociation, cannot be excluded. This residual desensitization is not unique to the toxin and was reported for AMPA receptors in complex with CTZ, and those carrying single-point mutations that block desensitization (LY and Lurcher mutations; Zhang et al., 2017). Even though the main mechanism of desensitization in AMPA receptors proceeds via the rupture of the LBD dimers (Sun et al., 2002), block of desensitization through mutations in the LBD-TMD linkers is a well-established concept (Yelshansky et al., 2004). This observation implies that desensitization processes acting through the linkers alone are possible.

We observed roughly the same four open levels for GluA2, either with CTZ, CII toxin, and/or (R, R)-2b bound, just like for freely-desensitizing AMPA receptors (Zhang et al., 2008). Receptors in complex with CII toxin shut frequently, gave a flat occupancy distribution across the three smallest amplitude sublevels, and rarely visited the maximum open level. Comparison of the toxin with other desensitization blockers, CTZ, and the more potent (R, R)-2b (Kaae et al., 2007), showed that frequent closures and a paucity of full-amplitude openings are characteristic for the toxin. With CII toxin bound, the receptors visited the closed state about 10-fold more frequently than with (R, R)-2b bound. Different occupancies of the closed and fourth open level are the main mechanistic difference between the toxin and (R, R)-2b. A similar effect has also been reported for CTZ when compared to freely desensitizing receptors (Fucile et al., 2006; Zhang et al., 2017). There was no difference in open channel amplitudes, and the time constants describing the closed and open levels were similar for all conditions. These results indicate that (R, R)-2b is a better stabilizer of the fully open state because it allows the receptors to reach the fourth open level more frequently, primarily at the expense of the closed state. On the other hand, with the toxin bound, receptors struggled to reach the fully activated state as often. In our recordings, exposure to both toxin and (R, R)-2b resulted in toxin-like behavior, with frequent visits to the closed state and rare visits to open level 4 (Table 1).

**Figure 8.**
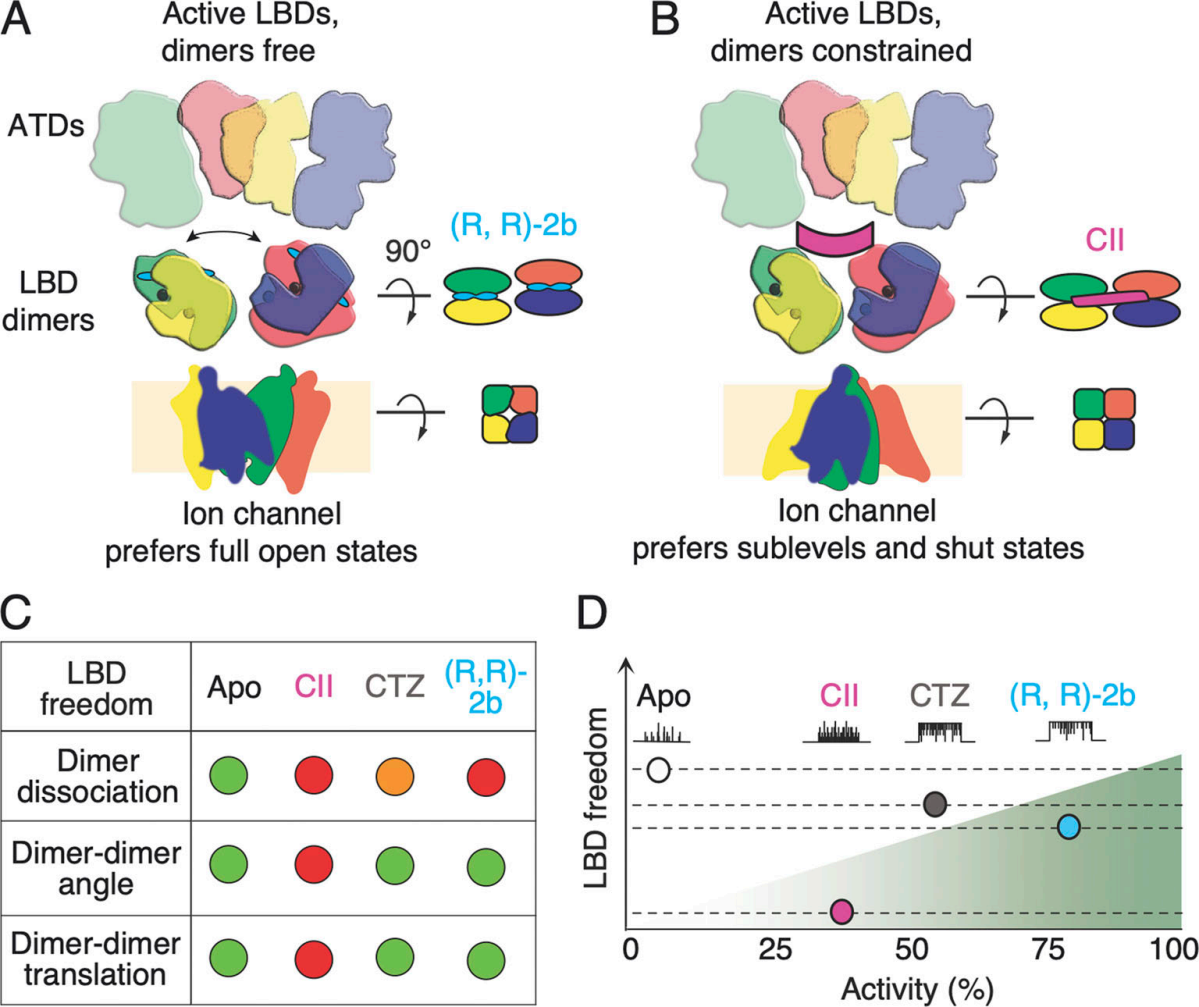
**Conformational freedom of ligand binding domain layer dictates activity of AMPA receptors. (A)** AMPA receptor subunits are color-coded, with green and yellow forming one LBD dimer, and blue and red another. AMPA receptors freely desensitize as long as the dimers are allowed to disassociate, resulting in very low activity of wild type receptors. Fixing the two monomers within each dimer, by CTZ or (R, R)-2b (cyan, two bars indicating its double-headed structure), blocks AMPA receptor desensitization and increases activity. **(B)** Preventing lateral movements and/or rotations of the LBD dimers, through binding of CII toxin (magenta) lowers the activity of the ion channel. **(C)** Summary of degrees of freedom of the LBD layer–green meaning high, red meaning low, and orange meaning intermediate. (R, R)-2b has lower *EC*_50_ when binding to LBD dimers than CTZ. **(D)** Fictive single-channel currents indicate increasing activity over four conditions, including apo from previous work (Carbone and Plested, 2012). (R, R)-2b supports the highest activity (*Q*_Frac_; Fig. 6) because it holds LBD dimers tightly together whilst allowing their free rotation and lateral translation to the optimal position(s).

In receptors exposed to both modulators, toxin and (R, R)-2b, toxin dominance over (R, R)-2b is expected due to its binding mode, i.e., due to the fact it locks LBD tetramer in a fixed conformation (see below and Fig. 8); however, the presence of (R, R)-2b did appear to produce some changes in the behavior. A small, but long time constant appeared in open level 4, which was never observed in receptors exposed to the toxin only (Fig. 7 and Table 2). Inspection of the recordings showed AMPA receptors exposed to both modulators had low and high activity modes, whose origins might be explained by different binding scenarios of CII and (R, R)-2b as explained in Fig. 5, B and C. Specifically, sequence of events whereby toxin dynamics allows (R, R)-2b to bind during the recording could conceivably produce observable switches between low activity (with toxin bound to both dimers) and high activity (with one dimer stabilized by (R, R)-2b and the other by partially-bound toxin). Reassociation of the toxin to both the dimers should re-establish low-activity mode characteristic of CII. Supporting this idea, the high activity mode could not be seen in receptors complexed by CII only. Different gating modes have been previously reported for AMPA receptors and linked to conditions that we did not vary in this study, namely glutamate concentration and voltage (Prieto and Wollmuth, 2010) and the presence of auxiliary subunits (Zhang et al., 2014). Thus, although the dominance of toxin over (R, R)-2b makes it hard to know with certainty that both modulators are bound on the time-scale of our single-channel recordings, the receptors exposed to both modulators did exhibit high activity behaviors, not seen in the CII-only condition.

Given that sublevel occupancy has been related to agonist binding to subunits that activate independently (Rosenmund et al., 1998) and that we worked in saturating glutamate (10 mM), this presents a conundrum about how CII works and how sublevel gating proceeds in general. The toxin binds at the opposite side of the LBD layer to the LBD-TMD linkers, and it seems unlikely that the toxin alters agonist binding (Chen et al., 2014) and somehow induces sublevels by altering occupancy by the agonist. Therefore, LBD inter-dimer angle and LBD dimer lateral displacement remain as mechanisms that permit the toxin to block desensitization, and at the same time to promote occupancy of a range of conductance states (Fig. 8). CII is a homodimer, with a flattened V-shape. When bound to a non-conductive channel, the toxin immobilizes the two LBD dimers at a fixed angle of ∼35°, similar to the angle that is observed in apo receptors (Chen et al., 2014; Dürr et al., 2014). When the channel is open (in structures without toxin), LBD dimers relax, adopting a ∼43° inter-dimer angle (Twomey et al., 2017). There is no structure of a toxin-bound AMPA receptor with an open pore, but the bound conformation of the toxin on the top of the LBD layer is unlikely to differ much between open and closed forms because toxin binding is so tight. Previous work showed that cross-links formed between the LBD dimers in AMPA receptors, which restrain lateral movements, lead to a decrease in receptor activity (Baranovic and Plested, 2018; Baranovic et al., 2016), and the action of the toxin seems related. This phenomenon appears largely irrespective of the nature of the bridge (disulfide or zinc bridges or flexible bifunctional cross-linkers) or its position. A similar observation has been reported for kainate receptors where disulfide bridges across the LBD dimers locked receptors in a semi-active conformation (Daniels et al., 2013). In contrast, a disulfide bridge at the LBD dimer interface locks NMDA receptors in a super-active state (Esmenjaud et al., 2019), but this must be considered in the context of standing inhibition by the ATD layer, which is absent in AMPA receptors. The opposing effects of CII toxin to block desensitization and also inhibit full activity, whilst still allowing full opening, suggest that, in the absence of auxiliary subunits, AMPA receptors sample a set of conformations when the channel pore is fully conducting. LBD layer dynamics, and a conformation distinct from that stabilized by the toxin, are key for high activity. In turn, this interpretation means that sublevels happen as a function of the LBD layer state, and are not necessarily just a consequence of LBD layer occupancy. Partial agonists and receptors saturated by glutamate that can desensitize, also allow sublevels (Carbone and Plested, 2012; Jin et al., 2003), and multiple overlapping sublevels are observed in other conditions (Coombs and Cull-Candy, 2021). Taking fractional activity as a metric, the toxin CII makes glutamate into a partial agonist, for example, compared to gating with (R, R)-2b. On the other hand, we saw that the main differences between different modulators were in the occupancy of closed and fully open levels, which could be taken to mean that sublevels are an integral feature of AMPA receptor gating in all conditions. The time constants for occupancy of sublevels O1, O2, and O3 (Fig. 7 and Table 2) were remarkably flat across all our conditions. More work is required to reconcile these different viewpoints.

We saw clear patch-to-patch heterogeneity in our recordings with all three blockers of desensitization. The extent of heterogeneity was highest in CTZ, followed by (R, R)-2b and then CII in accordance with their *EC*_50_ values (*EC*_50_ for CTZ: 5 μM, for (R, R)-2b: 0.4 μM, and for CII: 5 nM), It is, therefore, conceivable that some variability comes from modulator binding and unbinding during the recording. This interpretation is consistent with high and low activity modes observed for the receptors exposed to (R, R)-2b and CII, where the binding and unbinding dynamics lead to differential gating modes due to the starkly different abilities of (R, R)-2b and CII to stabilize the fully open channel, as shown by our analysis of idealized single-channel records.

Toxins have been used extensively as tools to study ion channel function (Kalia et al., 2015) and our work shows that the very low *EC*_50_ of the toxin should allow it to be used for experimental applications similar to antibodies, but without the associated bulk. CII is specific for AMPA receptors. It can bind to any of its subunit types (GluA1-4), but does not have an effect on the closely related kainate (GluK2) or NMDA receptors (GluN1/GluN2A) or GABA-A receptors (Walker et al., 2009). The toxin also blocks desensitization of native, likely heteromeric, AMPA receptors, which is unsurprising given that GluA2 residues interacting with the toxin are conserved across all four subunits (Chen et al., 2014). The observation that the toxin actually inhibits the maximum activity provides insight into how the toxin works in vivo. The fact that inhibition of desensitization alone is enough to generate a large excitotoxic current, even in the face of reduced activity, implies that CII exploits a large extrasynaptic receptor pool. Therefore, a key physiological role of desensitization is to mask the potentially deadly capacity of this receptor pool for generating depolarizing current. The initial report about CII (Walker et al., 2009) estimated the *EC*_50_ to be 67 nM. This value was an underestimation, likely due to the lack of time to equilibrate in the oocyte recording system. The low *EC*_50_ we determined with overnight incubation (5 nM) indicates that the toxin should tolerate mutations and derivatizations that would diversify its functionality whilst still binding well to AMPA receptors. Congruent with this tight binding, the toxin stays bound to GluA2 homomeric receptors for an average of 10 min. Due to its small size and unique binding site, it leaves the overall dimensions of the receptor unchanged and extracellular domains free to interact with other synaptic proteins, unlike antibodies or even Fab fragments labeling the ATDs (Giannone et al., 2010; Zhao et al., 2019). Although further characterization of the toxin is needed, such as possible effects of different auxiliary proteins, these properties suggest that the toxin has the potential as a tool for investigating native AMPA receptors.
